# Neutrophil-mediated myocardial ischemia–reperfusion injury: mechanisms and potential therapeutic targets

**DOI:** 10.3389/fimmu.2026.1821747

**Published:** 2026-09-29

**Authors:** Wenna Xu, Zhaoshan Zhang, Yuxin Wei, Zhongquan Zhou, Jiawei Guo

**Affiliations:** 1 Department of Cardiology, The First Affiliated Hospital of Yangtze University, Jingzhou, China; 2 Department of Pharmacology, School of Medicine, Yangtze University, Jingzhou, China

**Keywords:** immunomodulation, myocardial ischemia–reperfusion injury, neutrophil extracellular traps, neutrophils, therapeutic targets

## Abstract

Although reperfusion therapy improves the prognosis of acute myocardial infarction, myocardial ischemia–reperfusion injury continues to limit its clinical benefits, with neutrophil-driven inflammatory cascades representing a central mechanism of injury amplification. This article delineates the molecular event chain of neutrophils from recruitment to effector execution: activated neutrophils damage cardiomyocytes through three principal pathways—respiratory burst, degranulation, and neutrophil extracellular traps formation—thereby establishing a positive feedback loop that exacerbates inflammatory amplification. On this basis, we summarize therapeutic strategies targeting neutrophil migration, neutrophil extracellular traps formation, intracellular signaling pathways, mitochondrial homeostasis, immune phenotypes, and targeted drug delivery systems.

## 1 Introduction

Acute myocardial infarction is one of the leading cardiovascular diseases worldwide in terms of mortality and disability, and its pathological basis is persistent myocardial ischemia and cellular necrosis caused by acute coronary artery occlusion (1).At present, reperfusion strategies such as percutaneous coronary intervention and thrombolytic therapy have become standard treatment modalities, significantly reducing mortality and improving cardiac functional outcomes by rapidly restoring blood flow in the infarct-related artery (2, 3). However, restoration of blood flow is not a process of pure benefit. A large body of research has confirmed that while the ischemic myocardium regains oxygen supply, a series of secondary injury responses are simultaneously triggered, known as myocardial ischemia–reperfusion injury (MIRI) (4). This process is characterized by further cardiomyocyte death, microcirculatory perfusion disturbances, edema formation, and amplification of inflammatory responses, which to some extent counteract the protective effects of reperfusion therapy (5). Therefore, limiting secondary injury while ensuring vascular recanalization has become an important research focus in the cardiovascular field.

Among the various mechanisms contributing to MIRI, inflammation is considered a key link connecting ischemic injury with reperfusion-extended damage. In the early phase of reperfusion, oxidative stress is enhanced, calcium overload and mitochondrial dysfunction are rapidly initiated, and these processes interact with inflammatory responses to collectively exacerbate structural and functional myocardial damage (6). In this inflammatory network, neutrophils, as important components of the innate immune system, are widely recognized as one of the earliest and most abundant effector cells infiltrating the injured myocardium after reperfusion (7). Studies have shown that neutrophils are rapidly recruited to the ischemic region within minutes to hours after reperfusion, and their migration depends on selectin- and integrin-mediated adhesion and transendothelial migration (8). After entering myocardial tissue, activated neutrophils directly damage cardiomyocytes and vascular endothelial cells by releasing large amounts of reactive oxygen species, proteolytic enzymes, and pro-inflammatory cytokines, further amplifying the inflammatory cascade (8). Mechanistic studies indicate that neutrophil-mediated oxidative stress and activation of inflammatory signaling pathways are important driving factors for infarct expansion and deterioration of cardiac function (7). In addition, the formation of neutrophil extracellular traps provides a new molecular basis for understanding inflammatory amplification and microvascular obstruction during reperfusion (9). Clinical and experimental studies have found that neutrophil extracellular traps(NETs) significantly increase after acute myocardial infarction reperfusion and are closely associated with the no-reflow phenomenon and infarct size expansion; clearance of NETs can improve microcirculatory perfusion and promote recovery of cardiac function (10). Meanwhile, multiple animal studies have demonstrated that depletion of neutrophils or inhibition of their recruitment and activation can significantly alleviate reperfusion-related myocardial injury (11).

In summary, neutrophils play a critical role in the early inflammatory regulation of MIRI and contribute to myocardial damage through multiple mechanisms, including oxidative stress, degranulation, and NET formation (NETosis). This review systematically summarizes the molecular mechanisms underlying neutrophil recruitment, activation, and effector functions in MIRI, with particular emphasis on therapeutic strategies targeting neutrophil migration, NETosis, intracellular signaling pathways, mitochondrial function, and targeted delivery systems. Furthermore, by considering the functional heterogeneity of neutrophils, we discuss the challenges and future directions of precision intervention, providing a theoretical basis for the development of neutrophil-targeted therapies in MIRI.

MIRI can occur in various clinical settings. In this review, MIRI primarily refers to myocardial injury resulting from the restoration of coronary blood flow after acute myocardial infarction, with particular emphasis on ST-segment elevation myocardial infarction treated with reperfusion strategies such as percutaneous coronary intervention or thrombolysis. MIRI can also occur in other clinical settings, including cardiopulmonary bypass and cardiac surgery, but the ischemic conditions, reperfusion procedures, systemic inflammatory responses, and perioperative environment in these settings may differ from those associated with post-myocardial infarction reperfusion. Therefore, the mechanisms discussed in this review are primarily considered in the context of post-myocardial infarction coronary reperfusion, and their relevance to MIRI occurring in other clinical settings should be evaluated in light of the specific clinical context.

## 2 Recruitment and infiltration of neutrophils during reperfusion

### 2.1 Generation of early inflammatory signals

Within minutes to hours after restoration of myocardial blood flow, cellular structural damage accumulated during ischemia is rapidly converted into inflammatory signaling events, initiating innate immune activation during the early phase of reperfusion. Necrotic or injured cardiomyocytes release a variety of damage-associated molecular patterns (DAMPs) into the extracellular space, including high-mobility group box 1, adenosine triphosphate (ATP), and mitochondrial DNA, thereby establishing the molecular basis for innate immune activation in the early phase of reperfusion (12).

These DAMPs trigger inflammatory cascades through recognition by pattern recognition receptors (PRRs). HMGB1 binds to Toll-like receptor 4 (TLR4) and the receptor for advanced glycation end products (RAGE), activating the NF-κB signaling pathway and promoting the transcription of pro-inflammatory cytokines and chemokines, thereby enhancing inflammatory cell recruitment signals (13). Meanwhile, extracellular ATP induces NLRP3 inflammasome assembly via the P2X7 receptor, leading to Caspase-1 activation and promoting the maturation and release of IL-1β and IL-18, which further amplify the local inflammatory microenvironment (14, 15). Due to its abundance of unmethylated CpG motifs, mitochondrial DNA can further enhance inflammatory signaling and promote functional activation of neutrophils through TLR9 and NLRP3 pathways (16). Therefore, the DAMP–PRR axis constitutes the central molecular platform for initiating inflammation in the early phase of reperfusion (17).

In addition to DAMP signaling, the complement system is activated almost simultaneously during reperfusion. The complement cleavage product C5a markedly enhances neutrophil chemotaxis, adhesion capacity, and reactive oxygen species production via C5a receptor 1 (C5aR1), and is closely associated with microvascular dysfunction, representing an important mechanism that exacerbates reperfusion injury (18). Notably, C5a receptor 2 (C5aR2) exerts bidirectional regulatory effects in this process. Experimental studies have shown that C5aR2 confers protective effects by limiting excessive neutrophil infiltration mediated by C5aR1; deletion of C5aR2 aggravates inflammatory injury, whereas the C5aR1 antagonist PMX53 alleviates tissue damage, suggesting that excessive C5aR1 activation is a key pathogenic factor (18). The dynamic balance between C5aR1 and C5aR2 reflects the complex pro-inflammatory and regulatory roles of the complement system.

Driven by the above signaling cascades, IL-1β and TNF-α levels rapidly increase in myocardial tissue during the early phase of reperfusion. These cytokines not only further amplify local inflammatory responses but also induce endothelial phenotypic transformation, upregulate adhesion molecule expression, and impair barrier function (19, 20). IL-1β is considered a key upstream regulator of reperfusion inflammation; through a Caspase-1–dependent mechanism, it amplifies inflammatory signaling and disrupts mitochondrial homeostasis, thereby aggravating myocardial injury (20). TNF-α participates in regulating inflammatory cascades and apoptotic processes, further reinforcing the tissue-damaging microenvironment (19). Therefore, during the early phase of reperfusion, the release of DAMPs, complement activation, and the production of inflammatory mediators collectively constitute the initiating signals for neutrophil recruitment, establishing an inflammatory foundation for subsequent endothelial activation, adhesion, and tissue infiltration.

### 2.2 Endothelial activation and neutrophil recruitment

With the accumulation of early inflammatory signals, vascular endothelial cells gradually transition from a quiescent state to a pro-inflammatory phenotype, followed by a sequential process involving neutrophil rolling, adhesion, transendothelial migration, and chemotactic infiltration. TNF-α and IL-1β induce the upregulation of adhesion molecules such as selectins and ICAM-1 and alter endothelial barrier function, thereby creating conditions conducive to inflammatory cell recruitment (20, 21). During the initial phase following endothelial activation, P-selectin is rapidly translocated to the cell surface from Weibel–Palade bodies, whereas E-selectin is transcriptionally upregulated in response to inflammatory stimulation. Together, they mediate the rolling and deceleration of neutrophils under shear stress, laying the foundation for subsequent integrin-dependent firm adhesion (22).

Following the initial selectin-mediated rolling process, As local chemokine concentrations increase, G protein–coupled receptors on the surface of neutrophils are activated, inducing conformational changes in β2 integrins and enhancing their affinity for ICAM-1 (23). Firm adhesion mediated by LFA-1 and Mac-1 enables neutrophils to stably attach to the endothelial surface and initiate the process of transendothelial migration (21). Previous animal studies have shown that blocking the LFA-1/ICAM-1 interaction significantly improves cardiac functional recovery after reperfusion and attenuates tissue injury, further demonstrating that β2 integrin–dependent firm adhesion represents a critical inflammatory checkpoint in MIRI (24). Among β2 integrin family members, Mac-1 (CD11b/CD18) is markedly upregulated in ischemia–reperfusion models. Its activation not only enhances cellular adhesion but also amplifies inflammatory responses and promotes myocardial remodeling through signaling pathways including NF-κB, Bax, Caspase-3, and TGF-β/Smad2/3 (25). Notably, in renal ischemia–reperfusion models, Mac-1 has been reported to promote NETosis via an ERK-dependent mechanism, thereby exacerbating the inflammatory cascade (26). However, whether this mechanism similarly operates in the context of myocardial ischemia–reperfusion remains to be further experimentally validated. Following the establishment of firm adhesion, the later stage of transendothelial migration depends on the ability of neutrophils to efficiently traverse endothelial junctions. The endothelial metalloproteinase ADAM10 mediates cell detachment by cleaving the extracellular domain of ICAM-1; this process constitutes the rate-limiting step of migration, and inhibition of ADAM10 significantly prolongs transendothelial migration time (27). Through this mechanism, neutrophils complete their spatial transition from the vascular lumen to the interstitial tissue.

After completing adhesion and transendothelial migration, neutrophils undergo directed migration along chemokine concentration gradients formed within the injured region, which represents a key determinant of inflammatory lesion infiltration intensity (7). Among the CXC chemokine family, members containing the ELR motif (ELR^+^) are core drivers of this process. CXCL8 is markedly upregulated under inflammatory conditions and promotes accumulation at inflammatory sites by enhancing directional migratory capacity (28). CXCL5 is also significantly increased in ischemia–reperfusion models and aggravates tissue injury by enhancing neutrophil recruitment (29, 30).The effects of these chemokines primarily depend on CXCR1 and CXCR2 expressed on the surface of neutrophils, among which CXCR2 is considered the key receptor mediating neutrophil migration from the bloodstream to inflammatory foci. Upon receptor activation, downstream signaling pathways such as PI3K/Akt are initiated, inducing cytoskeletal rearrangement, polarity establishment, and pseudopod formation, thereby enabling efficient directed migration along the chemotactic gradient (28). In addition, angiotensin II can enhance inflammatory cell infiltration by upregulating CXCL8 expression, suggesting that neurohumoral factors also participate in regulating the chemotactic network (31).

Overall, neutrophil recruitment is a highly coordinated and temporally regulated process. Reperfusion-induced DAMPs release and complement activation initiate early inflammatory signaling, which subsequently promotes endothelial activation and the expression of adhesion molecules. Neutrophils then undergo sequential steps of rolling, firm adhesion, and transendothelial migration, and ultimately migrate toward the injured myocardium under the guidance of the CXCL8/CXCL5–CXCR1/2 chemokine axis.

Following myocardial ischemia–reperfusion, damaged cardiomyocytes, resident macrophages, and endothelial cells release damage-associated molecular patterns (DAMPs) and pro-inflammatory cytokines, which induce the expression of P-selectin and E-selectin on vascular endothelial cells, thereby initiating the neutrophil recruitment cascade. Circulating neutrophils interact with endothelial selectins via PSGL-1 expressed on their surface, mediating leukocyte rolling. Subsequently, chemokines bind to G protein–coupled receptors on neutrophils, triggering conformational changes and activation of integrins (LFA-1/Mac-1) through inside-out signaling. Activated integrins then bind with high affinity to ICAM-1 on endothelial cells, resulting in firm adhesion. Neutrophils subsequently undergo transendothelial migration across the endothelial barrier and infiltrate the myocardial tissue. Infiltrating neutrophils releases reactive oxygen species (ROS), proteolytic enzymes, and various pro-inflammatory mediators, thereby amplifying local inflammatory responses, promoting microvascular dysfunction, and ultimately exacerbating reperfusion injury (**Figure 1**).

**Figure 1:**
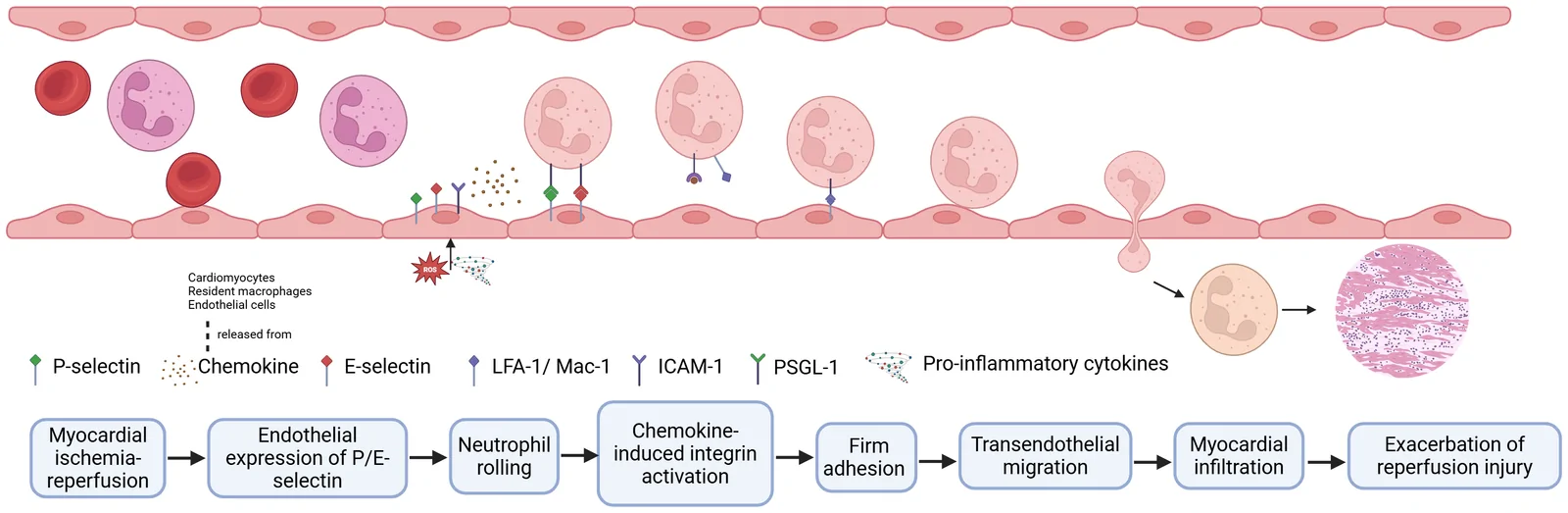
Molecular mechanisms of neutrophil recruitment in myocardial ischemia–reperfusion injury.

## 3 Mechanisms of neutrophil-mediated myocardial injury

MIRI results from the interplay of multiple factors, among which neutrophil recruitment, activation, and effector molecule release play critical roles in tissue damage. Neutrophils exacerbate cardiomyocyte injury and microvascular dysfunction through mechanisms including respiratory burst, degranulation, and NETosis.

### 3.1 Respiratory burst and oxidative stress

During the early stage of reperfusion, restoration of oxygen supply rapidly disturbs the redox balance within ischemic myocardium. Excessive production of ROS, together with impaired endogenous antioxidant defenses, triggers a phenomenon often referred to as the reoxygenation burst. This oxidative environment facilitates inflammatory cell recruitment and activation, creating favorable conditions for subsequent neutrophil-mediated tissue injury (32).

As inflammation progresses, neutrophils accumulate in the ischemic myocardium in response to chemokines, complement activation, and DAMPs. These stimuli induce the respiratory burst, a hallmark of neutrophil activation (33). Central to this process is the assembly of the NADPH oxidase 2 (NOX2) complex. Cytosolic NOX2 subunits translocate to the plasma membrane and associate with the membrane-bound gp91^phox/p22^phox heterodimer, forming an active enzyme complex that catalyzes the conversion of NADPH to superoxide anion (O_2_^-^) (32). Superoxide is rapidly dismutated into hydrogen peroxide (H_2_O_2_) by superoxide dismutase (SOD), after which myeloperoxidase (MPO) converts H_2_O_2_ into highly reactive oxidants such as hypochlorous acid (HOCl). These oxidants substantially increase the oxidative capacity of activated neutrophils (7). Unlike antimicrobial responses directed against pathogens, neutrophil activation during sterile inflammation results in the uncontrolled release of ROS into the myocardial interstitium and vascular endothelium. The resulting oxidative microenvironment amplifies local inflammation and accelerates tissue injury (34). ROS contribute to tissue damage through multiple mechanisms: (1) inducing lipid peroxidation and disrupting membrane integrity by directly attacking membrane phospholipids; (2) oxidizing protein thiol groups and critical catalytic residues, thereby impairing protein function and cellular metabolism; and (3) causing oxidative DNA damage and activating DNA damage response pathways, ultimately leading to cellular dysfunction (35). In addition, NOX2-derived ROS can propagate into surrounding tissues, expanding the extent of oxidative injury and sustaining local inflammatory responses.

At the level of intracellular signaling, neutrophil-derived ROS activate multiple pro-inflammatory pathways, including MAPK, JNK, and NF-κB signaling, thereby promoting the transcription of inflammatory cytokines such as TNF-α and IL-1β (36). Activated NF-κB further augments TNF-α production, whereas TNF-α stimulates the expression of chemokines and adhesion molecules, promoting additional leukocyte recruitment (11, 37). In turn, TNF-α and IL-1β reinforce each other’s production, establishing a self-amplifying ROS–NF-κB–cytokine signaling loop that sustains inflammatory responses during MIRI (38). Thus, NOX2-dependent ROS generation serves as a critical link between oxidative stress and inflammatory amplification during the early phase of MIRI.At the vascular level, ROS released by activated neutrophils also impair endothelial function by reducing nitric oxide (NO) bioavailability. Specifically, superoxide generated by NOX2 reacts rapidly with NO to form peroxynitrite (ONOO^-^), thereby attenuating endothelium-dependent vasodilation and promoting nitrosative stress (39). Meanwhile, ROS disrupt endothelial intercellular junctions, compromise vascular barrier integrity, and increase vascular permeability, facilitating inflammatory cell extravasation into injured myocardium (40). These vascular changes further intensify local inflammatory responses.

These findings place the respiratory burst at the center of neutrophil-mediated inflammatory injury during MIRI. Rather than representing only an early activation event, NOX2-derived ROS initiate a cascade of downstream responses, including degranulation and NETosis. By coupling oxidative stress with inflammatory signaling, respiratory burst sustains neutrophil effector functions and drives the progression of myocardial injury throughout the reperfusion phase (32).

### 3.2 Degranulation and protease release

Concurrent with the respiratory burst, another key effector mechanism of neutrophils—the degranulation response—is also initiated. Neutrophil cytoplasm contains various functional granules, including primary, secondary, and tertiary granules. Upon inflammatory stimulation, these granules release large amounts of proteases and oxidases through exocytosis, constituting a direct effector mechanism for structural damage in MIRI.

#### 3.2.1 MPO-mediated oxidative damage

MPO is the core enzyme of primary granules, catalyzing the production of HOCl from H_2_O_2_ generated during the respiratory burst (41). HOCl oxidizes protein thiols, lipid double bonds, and DNA bases, leading to protein conformational changes and functional disruption (42).During MIRI, MPO expression and activity are significantly elevated, and their levels positively correlate with infarct size and cardiac functional decline (43). HOCl not only exacerbates local oxidative stress but also amplifies the effects of the respiratory burst, extending oxidative damage into the extracellular microenvironment (42). MPO and the respiratory burst exhibit a synergistic amplification: the respiratory burst provides H_2_O_2_ substrate, while HOCl further activates neutrophils to promote additional ROS generation. MPO deposits on the endothelial surface, aggravating microvascular dysfunction by promoting LDL oxidation, enhancing inflammatory cell adhesion, and amplifying chemotactic signaling. Endothelial oxidative injury impairs vasodilation and increases permeability, creating a vicious cycle of “endothelial damage → inflammatory recruitment → more MPO deposition” (44).

#### 3.2.2 NE- and MMP-9–mediated extracellular matrix degradation

Neutrophil elastase (NE) is the most abundant serine protease in primary granules. During MIRI, released NE directly degrades extracellular matrix components such as elastin, collagen, and laminin, disrupting the mechanical support between cardiomyocytes and the matrix (33, 45). This destruction expands the necrotic border during the acute phase and interferes with repair during the subacute phase. NE-deficient mice exhibit reduced myocardial injury, decreased inflammatory infiltration, and improved cardiac function (46).

Matrix metalloproteinase-9 (MMP-9) primarily targets type IV collagen and the basement membrane. In the early phase of reperfusion, neutrophils rich in MMP-9 infiltrate the injured region, degrading the endothelial basement membrane, compromising its structural integrity, and promoting further inflammatory cell extravasation (47). Basement membrane disruption not only exacerbates acute inflammation but also affects later scar formation and ventricular remodeling. MMP-9 can also regulate the activity of inflammatory mediators such as IL-8, forming a positive feedback loop between sustained inflammation and ECM degradation (48). Recent studies show that MMP-9 can promote NETosis, worsening microvascular obstruction (49). MMP-9 deficiency significantly attenuates MIRI severity (50).

NE and MMP-9 form a synergistic proteolytic network: NE degrades the interstitial matrix, disrupting the extracellular scaffold of cardiomyocytes; MMP-9 damages the basement membrane barrier, promoting inflammatory extravasation. Together, they amplify tissue injury from both interstitial and vascular dimensions, and sustained ECM degradation weakens myocardial wall mechanics, driving adverse ventricular remodeling (47–50).

Degranulation products interact with inflammatory amplification in multiple ways: MMP-9 promotes inflammatory cell extravasation, MPO deposition continuously recruits inflammatory cells, and together with respiratory burst–mediated pro-inflammatory signals, they drive a positive feedback loop of “matrix degradation → inflammatory recruitment → more protease release” (48). In addition, NE and MPO are important components of NETs and contribute to subsequent inflammatory amplification by integrating into NET structures.

### 3.3 Neutrophil extracellular traps

MIRI is a complex pathological process driven by intertwined mechanisms, including oxidative stress, calcium overload, mitochondrial dysfunction, and dysregulated inflammation. The discovery of NETs has provided a new perspective for understanding MIRI. NETs are web-like structures released by neutrophils under extreme activation. They are not merely byproducts of inflammation, but critical hubs linking innate immunity, thrombosis, and tissue injury, playing an essential role in determining the severity and prognosis of MIRI.

#### 3.3.1 Molecular mechanisms of NETosis

During myocardial ischemia–reperfusion, the abrupt burst of ROS, together with the release of DAMPs and pro-inflammatory cytokines from injured cardiomyocytes, creates a highly inflammatory microenvironment that favors NETosis (51). Based on their initiating signals and molecular mechanisms, NETosis can generally be classified into two major forms: the classical ROS–PAD4-dependent pathway and inflammasome/GSDMD-associated NETosis.

The classical form of NETosis is primarily driven by the ROS–PAD4 signaling axis. ROS generated during reperfusion activate peptidylarginine deiminase 4 (PAD4), which catalyzes histone citrullination. This modification neutralizes the positive charge of histones, weakens histone–DNA interactions, and promotes chromatin decondensation (52).

The decondensed chromatin subsequently associates with granule proteins, including MPO, NE, and histones, forming the DNA-based scaffold of NETs. These structures are then released into the extracellular space through plasma membrane rupture or vesicle-mediated export (53). This process reflects a functional transition of neutrophils from conventional phagocytic antimicrobial activity toward extracellular inflammatory effector functions (54). Experimental studies of MIRI have demonstrated that excessive NETosis exacerbates cardiac damage, whereas citrullinated histone H3 has become a widely accepted biomarker for evaluating NETosis and its pathological significance (55).

Recent evidence suggests that, in addition to the classical ROS–PAD4-dependent pathway, inflammasome/GSDMD-mediated pyroptotic NETosis represents another important mechanism linking inflammatory signaling to NETosis. Unlike classical NETosis, this pathway is initiated by inflammasome activation and subsequent Gasdermin D (GSDMD) cleavage. Upon inflammasome activation, inflammatory caspases cleave GSDMD to generate the pore-forming N-terminal fragment (GSDMD-N), which inserts into the plasma membrane, increases membrane permeability, and facilitates chromatin extrusion and NET release (9, 56). GSDMD has emerged not only as a key executor of pyroptosis but also as a critical regulator of NETosis by promoting nuclear expansion, chromatin decondensation, and extracellular DNA release (57). Furthermore, both canonical and non-canonical inflammasome-associated caspase pathways participate in the regulation of NETosis through GSDMD. Beyond mediating NET release, the caspase–GSDMD axis also contributes to early nuclear membrane permeabilization, histone degradation, and chromatin remodeling (58, 59).

In MIRI, activation of the NLRP3 inflammasome drives inflammatory cell death through the caspase-1–GSDMD pathway while promoting the maturation and release of IL-1β and IL-18, thereby amplifying sterile inflammation and exacerbating myocardial injury (60). Accordingly, GSDMD-dependent pyroptotic NETosis is increasingly recognized as a critical mechanistic link connecting NLRP3 inflammasome activation, neutrophil effector functions, and tissue damage (61). These findings further suggest that targeting the NLRP3–GSDMD inflammatory axis may represent a promising therapeutic strategy for limiting aberrant neutrophil activation and interrupting inflammatory amplification during MIRI.

During myocardial ischemia–reperfusion, damaged cardiomyocytes release damage-associated molecular patterns (DAMPs) and pro-inflammatory cytokines, which activate neutrophils infiltrating the myocardial tissue. Activated neutrophils can generate NETs through two distinct pathways: lytic NETosis and non-lytic (vital) NETosis. In lytic NETosis, neutrophils undergo nuclear delobulation, disassembly of the nuclear envelope, and chromatin decondensation, ultimately leading to plasma membrane rupture. Decondensed chromatin decorated with granular proteins is then released into the extracellular space, accompanied by neutrophil death. In contrast, non-lytic NETosis involves vesicle-mediated transport of nuclear material and extracellular assembly of NETs without immediate cell lysis. In this process, neutrophils remain viable, retain certain effector functions such as phagocytosis, and continue to participate in the inflammatory response (**Figure 2**).

**Figure 2:**
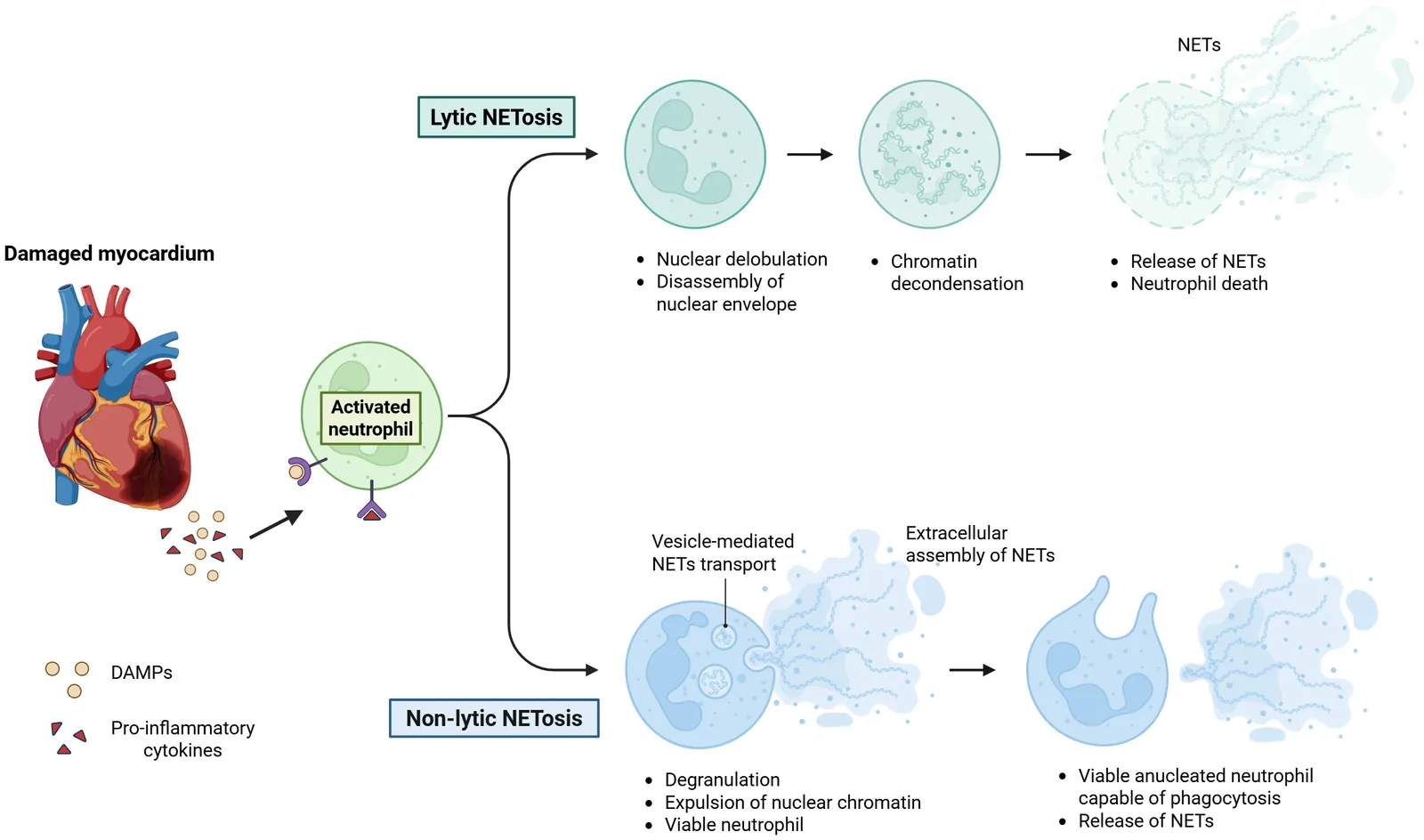
Mechanisms of neutrophil extracellular trap (NET) formation in myocardial ischemia–reperfusion injury.

#### 3.3.2 NETs and the microvascular “no-reflow” phenomenon

In certain patients, reopening of the epicardial coronary artery after reperfusion does not restore effective myocardial perfusion because of persistent microvascular obstruction, a condition referred to as the “no-reflow” phenomenon (62).

NETs aggravate microcirculatory dysfunction through combined structural and prothrombotic effects. The fibrous DNA networks deposit within capillary lumens, forming mechanical barriers that physically obstruct blood flow (63). Meanwhile, the DNA scaffold and histone components of NETs recruit platelets, erythrocytes, and fibrinogen, promoting microthrombus formation (64). NET-associated NE and MPO further enhance coagulation by activating tissue factor or directly interacting with coagulation factors, increasing thrombin generation, platelet aggregation, and fibrin cross-linking (53).

The interaction between mechanical obstruction and a hypercoagulable microenvironment result in sustained microvascular blockage. Consequently, myocardial tissue may remain ischemic even after reopening of the main coronary artery, thereby facilitating infarct expansion (9). Clinical studies have shown that plasma NET biomarkers in STEMI patients positively correlate with infarct size and thrombus burden (65).

#### 3.3.3 NET-mediated cytotoxic effects on cardiomyocytes

NETs directly injure cardiomyocytes through multiple interconnected mechanisms. Positively charged free histones bind electrostatically to negatively charged phospholipid bilayers of the cell membrane, insert into the membrane, disrupt structural integrity, and induce calcium influx, calcium overload, and mitochondrial dysfunction (66, 67). NETs components also function as DAMPs and are recognized by TLR2 and TLR4 on cardiomyocytes or immune cells, activating the NF-κB pathway and initiating pro-inflammatory gene transcription (68).

In addition, NETs activate the NLRP3 inflammasome, promoting caspase-1–dependent maturation and release of IL-1β and inducing pyroptotic cell death (69, 70). NETs further stimulate complement activation, leading to membrane attack complex (C5b-9) formation on cardiomyocyte membranes and aggravating lytic cell death (71).Experimental evidence supports the inflammatory amplification role of NETs. Inhibition of NLRP3 reduces IL-1β and IL-18 release and alleviates reperfusion injury (72). Neutrophil-intrinsic NLRP3 activation promotes IL-1β production and NETs deposition, forming a positive feedback loop characterized by enhanced NETosis and sustained inflammatory activation (73, 74).

Building on the aforementioned mechanisms, recent studies have increasingly recognized that neutrophil-mediated inflammatory injury is not driven by respiratory burst, degranulation, and NETosis as independent effector programs, but rather results from a dynamic regulatory network involving interactions among distinct functional modules. ROS generated during the respiratory burst not only induce oxidative injury but also promote the activation of NETosis-related signaling pathways. Meanwhile, granule proteins released during degranulation, including MPO and NE, contribute to local tissue damage and serve as important components of NET structures. Once formed, NETs further enrich oxidases, proteases, and inflammatory mediators, thereby enhancing oxidative stress, endothelial injury, and inflammatory cell recruitment, while promoting additional ROS generation and degranulation responses. Therefore, respiratory burst, degranulation, and NETosis do not represent a simple linear cascade, but rather form a mutually reinforcing positive-feedback regulatory network that collectively drives early inflammatory injury during MIRI.

It is noteworthy that the biological effects of NETs in MIRI are not entirely fixed but may exhibit stage-dependent and context-dependent characteristics. During the acute phase of reperfusion, excessive NETosis primarily aggravates tissue injury by promoting microvascular obstruction, inflammatory amplification, and cardiomyocyte death. However, as inflammation transitions toward resolution and tissue repair, the effects of NETs may be influenced by the local inflammatory microenvironment and NET clearance efficiency, and their potential roles in tissue repair remain to be further elucidated. Conversely, persistent NET accumulation or impaired NET clearance may sustain chronic inflammation, thereby promoting myocardial fibrosis and adverse ventricular remodeling. Thus, the biological functions of NETs may dynamically change with disease progression and alterations in the local inflammatory microenvironment, highlighting that future NET-targeted interventions should consider therapeutic timing and disease stage rather than completely blocking NETosis (8, 9, 75).

Overall, NETs are not merely passive byproducts of neutrophil activation but represent a critical pathological platform integrating oxidative stress, protease release, thrombosis, and immune-inflammatory amplification. During MIRI, NETs convert early neutrophil activation into sustained tissue injury through multiple mechanisms, including microvascular obstruction, cardiomyocyte toxicity, and inflammatory positive feedback, while also contributing to subsequent adverse ventricular remodeling. Therefore, NETs represent not only an important pathogenic mechanism in MIRI but also a potential therapeutic target with a defined therapeutic window.

## 4 Therapeutic targets

### 4.1 Targeting neutrophil recruitment and adhesion

Neutrophil infiltration into injured myocardium represents a pivotal initiating event in MIRI. Intervening in the recruitment process can interrupt the inflammatory cascade at its source.

#### 4.1.1 Inhibition of Rac-1 signaling

The dual-function inhibitor Nexinhib20 antagonizes the binding of Rac-1 to GTP, thereby suppressing Rac-1 activation. This simultaneously blocks neutrophil exocytosis and β2-integrin activation. In experimental models of MIRI, post-ischemic administration significantly reduced neutrophil recruitment and decreased infarct size, demonstrating promising translational potential (76).

#### 4.1.2 Inhibition of the chemokine receptor CXCR2

Chemokine signaling is a central driver of directed neutrophil migration. Studies have shown that the CXCR2 receptor inhibitor Navarixin significantly improves cardiac function, attenuates myocardial injury and fibrosis, and reduces inflammatory cytokine expression by suppressing neutrophil recruitment to infarcted myocardium (77). Transcriptomic analysis further revealed that its mechanism involves modulation of innate immune responses and chemokine signaling pathways, effectively reducing neutrophil chemotaxis (77).

Because chemokines exhibit relatively specific cellular targets, this finding provides important theoretical support for designing targeted intervention strategies to optimize cardiac repair and prevent adverse ventricular remodeling (78). In addition, MKEY, a compound that disrupts the CCL5–CXCR4 interaction, significantly reduces infarct size following myocardial ischemia/reperfusion in mice, preserves cardiac function, suppresses neutrophil and macrophage infiltration, and decreases NETosis (79).

#### 4.1.3 Targeting endothelial adhesion molecules

Vascular endothelial activation is an upstream event in neutrophil recruitment. The vascular leakage blocker CU06–1004 enhances vascular integrity and downregulates endothelial VCAM-1 expression, thereby inhibiting neutrophil and macrophage infiltration. This leads to reduced myocardial necrosis and edema and improved cardiac function. A single high-dose treatment demonstrated superior long-term efficacy compared to repeated low-dose administration at eight weeks after ischemia–reperfusion, highlighting its potential as an adjunctive reperfusion therapy (80).

Based on this strategy, researchers have developed endothelial-targeted, ROS-responsive nanocomposites capable of delivering VCAM-1 siRNA and dexamethasone. Under excessive ROS conditions, these nanocomposites release their therapeutic payload, enabling precise intervention in inflammation-activated endothelium, inhibiting neutrophil migration and adhesion, and enhancing anti-inflammatory effects (81).

### 4.2 Targeting NETosis and neutrophil effector functions

NETs are web-like structures released upon neutrophil activation that can directly injure cardiomyocytes and amplify inflammatory responses. In recent years, significant progress has been made in identifying key regulatory molecules involved in NETosis and exploring their clinical translational value. These therapeutic targets are mainly distributed across three levels: intrinsic neutrophil signaling pathways, post-translational modification mechanisms, and clinically accessible NET inhibitors.

At the level of intrinsic neutrophil signaling pathways, the MLK3–C/EBPβ–CRAMP axis has been identified as a critical driver of NETosis. MLK3 promotes CRAMP expression through activation of C/EBPβ, thereby facilitating NET generation. Neutrophil-specific MLK3 deficiency or pharmacological inhibition with the MLK3 inhibitor CEP-1347 significantly reduces infarct size, improves cardiac function, and decreases NETosis in murine models of myocardial infarction. Clinical data further indicate that phosphorylated MLK3 levels in circulating neutrophils from patients with acute myocardial infarction positively correlate with myocardial injury biomarkers (82).

In contrast to the pro-NET effect of MLK3 signaling, activation of the TRAIL–DR5 pathway suppresses NETosis through downstream inhibitory signaling. Studies have shown that inhibition of TRAIL–DR5 signaling reduces PAD4 expression in neutrophils, thereby decreasing NETosis, lowering adhesion molecule and chemokine release, attenuating neutrophil infiltration, and ultimately alleviating MIRI (83). Together, these pathways illustrate the finely balanced intrinsic signaling network that positively and negatively regulates NETosis in neutrophils.

At the level of post-translational modification, emerging regulatory mechanisms have also been implicated in NET control. Lactylation of neutrophil S100a9 at lysine 26 (S100a9K26la) is markedly elevated in acute myocardial infarction patients and in murine ischemia–reperfusion models. Lactylated S100a9 translocates to the nucleus, promotes neutrophil migration and cardiac recruitment, and induces cardiomyocyte mitochondrial damage and cell death through NET release. This modification is catalyzed by DLAT, and inhibition of DLAT blocks S100a9 lactylation, reduces neutrophil infiltration, and improves cardiac function. Clinically, plasma S100a9K26la levels positively correlate with adverse outcomes in patients (84).

At the level of clinically accessible NET inhibitors, several established drugs and novel agents have demonstrated therapeutic potential based on these mechanistic insights. Colchicine suppresses the S100A8/A9 pathway, reducing bone marrow neutrophil proliferation, recruitment, and NETosis, thereby decreasing cardiomyocyte apoptosis and oxidative stress and alleviating microvascular obstruction following PCI in STEMI patients (85). PAD4, a key enzyme in NETosis, has also been implicated in exacerbating hypoxia/reoxygenation injury in cardiomyocytes. The PAD4-specific inhibitor GSK484 preserves myocardial ATP levels and mitigates cellular injury (86).Collectively, these findings suggest that targeting NETosis and PAD4 offers a promising multi-target therapeutic strategy for mitigating MIRI.

In addition to NETosis, NOX2-dependent oxidative responses in neutrophils represent another major contributor to the early inflammatory injury observed in MIRI. Accordingly, targeting neutrophil-derived ROS and their downstream oxidative products has emerged as a promising therapeutic strategy. Thiocyanate (SCN^-^) competitively serves as an alternative substrate for MPO, thereby reducing the generation of HOCl, limiting infarct size, and improving left ventricular function (87). Naturally occurring polyphenolic compounds have also shown considerable potential in mitigating oxidative inflammation. Gallic acid reduces malondialdehyde (MDA) levels, attenuates inflammatory responses, and restores the Bcl-2/Bax balance, ultimately reducing myocardial infarct size (88). Similarly, baicalein enhances SOD activity while decreasing MDA and MPO levels, thereby alleviating cardiomyocyte apoptosis and inflammatory injury (89).

### 4.3 Targeting intracellular signaling pathways

#### 4.3.1 AMPK signaling pathway activators

AMPK is a key energy-sensing molecule in MIRI and plays a protective role in regulating energy metabolism, oxidative stress, mitochondrial homeostasis, and inflammatory responses (90). During ischemia–reperfusion, energy imbalance and ROS bursts suppress AMPK activity, and pharmacological activation of AMPK is therefore considered an important cardioprotective strategy (91).

Gemfibrozil, administered at 100 mg/kg prior to ischemia in a murine ischemia-reperfusion injury model, significantly improves cardiac function, reduces infarct size, and enhances myocardial antioxidant and anti-apoptotic capacity. Its protective effects depend on maintaining phosphorylated AMPK levels, as AMPK inhibition reverses these benefits (92). The selective AMPK activator MK-3903 similarly improves left ventricular systolic function, reduces cardiomyocyte apoptosis and inflammation, and promotes mitochondrial biogenesis and homeostasis through the AMPK–PGC-1α pathway, while inhibition of PGC-1α abolishes these protective effects (93). In addition, lactoferrin attenuates oxidative stress and inflammation and improves cardiomyocyte survival in doxorubicin- and IRI-induced myocardial injury models by activating AMPK and suppressing ferroptosis pathways (94).

#### 4.3.2 NLRP3 inflammasome inhibitors

The NLRP3 inflammasome is a central inflammatory complex mediating pyroptosis in MIRI. Its activation induces caspase-1 cleavage, GSDMD activation, and IL-1β/IL-18 release, thereby exacerbating local inflammation and expanding myocardial injury (95). Therefore, inhibition of NLRP3 inflammasome activation has emerged as an important therapeutic strategy in recent years.

During ischemia–reperfusion, oxidative stress, ROS accumulation, and TXNIP upregulation promote NLRP3 inflammasome activation. Studies have shown that geniposide activates AMPK, reduces ROS levels, and downregulates TXNIP expression, thereby inhibiting the interaction between TXNIP and NLRP3, suppressing inflammasome activation and pyroptosis, reducing infarct size, and improving cardiac function (96).

Multiple natural products and small-molecule inhibitors also exhibit cardioprotective effects. Puerarin significantly reduces infarct size, decreases caspase-1 and GSDMD expression, and improves mitochondrial function in animal models. Its mechanism involves inhibition of NLRP3 inflammasome activation and activation of the NRF2/HO-1 antioxidant pathway (97). Panax notoginseng saponins suppress NLRP3 activation through modulation of the HMGB1/TLR4/NF-κB signaling axis, reduce infarct size, inflammatory cytokine release, and neutrophil infiltration, and inhibit pyroptosis. Combined administration with the NLRP3 inhibitor MCC950 further enhances its protective effects (98). Cinnamaldehyde pretreatment improves diastolic function, reduces infarct size, suppresses myocardial enzyme elevation and apoptosis, and attenuates oxidative stress and inflammation. Its mechanism involves inhibition of NLRP3 inflammasome activation and GSDMD-mediated pyroptotic signaling, thereby reducing IL-1β and IL-18 release (99). In addition, trimetazidine has been shown to attenuate MIRI by inhibiting the TLR4/MyD88/NF-κB/NLRP3 signaling pathway, thereby reducing GSDMD-N expression and suppressing pyroptotic cell death (100). Tongxinluo also exerts cardioprotective effects by inhibiting the NLRP3/caspase-1/GSDMD pyroptosis pathway, leading to reduced inflammatory cytokine release and neutrophil infiltration while preserving endothelial function and improving microvascular injury (101).

Recent studies have identified the cGAS–STING signaling pathway as a central hub linking DNA damage and inflammatory amplification in ischemia-reperfusion injury, which promotes NLRP3 inflammasome activation. STING inhibition or gene silencing significantly attenuates myocardial injury (102). In addition, neutrophil-derived Cathelicidin (Camp) is markedly upregulated in the early phase of ischemia-reperfusion injury and promotes IL-1β release via the TLR4/P2X7R/NLRP3 axis, amplifying inflammatory cascades. Camp deficiency alleviates inflammation and reduces infarct size (103).

Overall, therapeutic strategies targeting the NLRP3-associated inflammatory network can suppress inflammatory amplification during MIRI by modulating multiple upstream signaling pathways, including AMPK/TXNIP, TLR4/NF-κB, cGAS–STING, and TLR4/P2X7R/CAMP, thereby reducing caspase-1 activation, GSDMD-mediated pyroptosis, and the release of pro-inflammatory cytokines. In addition, the involvement of the NLRP3–GSDMD axis in pyroptotic NETosis further suggests that this pathway serves not only as a key regulator of inflammatory cell death but also as an important mechanistic link between neutrophil effector functions and myocardial tissue injury. Therefore, targeting the NLRP3–GSDMD inflammatory axis may represent a promising therapeutic strategy for limiting excessive inflammatory activation and alleviating MIRI.

#### 4.3.3 Epigenetic regulation

In recent years, epigenetic regulation has been recognized as a key mechanism in MIRI, including DNA methylation, histone modifications, RNA methylation, and non-coding RNAs, all of which participate in the regulation of inflammation, apoptosis, and mitochondrial homeostasis (104).

Studies have shown that the SGLT2 inhibitor dapagliflozin improves cardiac function by downregulating EZH2, reducing H3K27me3 modification, and upregulating SIRT1, while simultaneously suppressing fibrosis and inflammation (105). miR-181b-5p is upregulated in MIRI, and its inhibition reduces cardiomyocyte apoptosis and infarct size by targeting AKT3/PIK3R3 and suppressing the PI3K/Akt pathway (106). Upregulation of the RNA methyltransferase METTL3 promotes pathological mitochondrial fission through the DNA-PKcs/Fis1 pathway; cardiomyocyte-specific deletion of METTL3 alleviates inflammation and neutrophil infiltration and reduces infarct size (107). Additionally, circular RNA circ_SMG6 exacerbates MIRI via the miR-138-5p/EGR1/TLR4/TRIF signaling axis, and EGR1 knockdown improves cardiac function, reduces infarct size, and inhibits cardiomyocyte apoptosis (108).

### 4.4 Targeting mitochondrial function and oxidative stress

Mitochondrial dysfunction and oxidative stress are closely interconnected and together constitute a central pathological basis of MIRI. During reperfusion, mitochondrial calcium overload, dissipation of the mitochondrial membrane potential, and disruption of the electron transport chain collectively impair mitochondrial function, leading to excessive production of mitochondrial reactive oxygen species (mtROS). Elevated mtROS further promotes mitochondrial permeability transition pore (mPTP) opening, cytochrome *c* release, and activation of downstream apoptotic cascades (109). As mitochondrial damage progresses, dysfunctional mitochondria become an additional source of ROS, establishing a self-perpetuating “ROS-induced ROS release” cycle that amplifies oxidative injury and accelerates cardiomyocyte dysfunction and death (34).

#### 4.4.1 Mitochondrial protective strategies

Given the high energy demand of the myocardium, mitochondria play a central role in maintaining ATP production and cardiac contractile function. Therefore, preserving mitochondrial structural integrity and functional homeostasis is essential for limiting MIRI (110). Current research mainly targets mitochondria at three levels: maintenance of calcium homeostasis, promotion of mitochondrial biogenesis, and improvement of mitochondrial dynamics balance.

Maintaining calcium homeostasis is critical for early intervention. 2-APB suppresses excessive activation of the mitochondrial calcium uniporter (MCU) by modulating the ITPR1/MCU pathway, thereby preserving mitochondrial calcium balance and attenuating myocardial injury (111). Excessive MCU activation can stimulate calpain and downregulate OPA1, leading to imbalance in mitochondrial dynamics; inhibition of this pathway restores homeostasis (112). Grpel2 protects mitochondrial stability by suppressing MCU-mediated calcium overload; its deficiency aggravates injury, which can be reversed by the MCU inhibitor Ru360 (113). HINT2 also improves mitochondrial fission and dysfunction by inhibiting MCU overactivation, while enhancing microcirculation and suppressing inflammation (114).

Promoting mitochondrial biogenesis enhances functional reserve capacity. Cannabidiol increases mitochondrial number and oxidative phosphorylation efficiency through activation of the SIRT1/PGC-1α axis, repairs myocardial structure, and suppresses inflammation and apoptosis (115). The endogenous peptide MOTS-c reduces infarct size, improves cardiac function, and enhances antioxidant and cytoprotective signaling, involving MAPK, mTOR, AMPK, and NRF2 pathways (116).

Improving mitochondrial dynamics is another important strategy. LTC restores mitochondrial morphology and membrane potential, reduces ROS and Ca²^+^ accumulation, and regulates fission/fusion proteins as well as NOX2/NOX4 expression, thereby achieving dynamic mitochondrial quality balance (117). Total saponins of Panax notoginseng reduce infarct and no-reflow areas by inhibiting the NR4A1/Mff/Drp1 pathway to suppress mitochondrial fission, while modulating VDAC1, HK2, cytochrome c, and caspase-9 to inhibit mitochondrial apoptosis (118).

#### 4.4.2 Oxidative stress intervention

Oxidative stress is not only a consequence of mitochondrial dysfunction but also a major driver of MIRI. During reperfusion, mitochondrial calcium overload and disruption of the electron transport chain markedly increase mtROS production. Excessive mtROS damages mitochondrial membranes and mitochondrial DNA, promotes cell death, and further impairs mitochondrial function, thereby establishing a self-perpetuating cycle of oxidative injury (6).

Current therapeutic strategies targeting oxidative stress mainly focus on limiting oxidative damage and reinforcing endogenous antioxidant defenses. Silibinin has been shown to inhibit the IKKα/IκBα/p65 signaling pathway, thereby improving cardiac function, reducing infarct size, and attenuating mitochondrial injury and cardiomyocyte apoptosis (119). Another promising approach is the activation of the Nrf2/HO-1 antioxidant pathway, which enhances the cellular antioxidant capacity. Bortezomib activates this pathway by upregulating Nrf2/HO-1 and increasing the expression of antioxidant molecules, including SOD1, CAT, and GSH, ultimately alleviating myocardial injury and improving cardiac function (120).

### 4.5 Targeting immune cell subsets and polarization

The function of immune cells is not uniform or static. Their heterogeneity and plasticity provide new perspectives for precision intervention in MIRI. Neutrophils and macrophages, as core executors of the inflammatory response, play decisive roles in balancing tissue injury and repair depending on their specific subsets and polarization states.

#### 4.5.1 Neutrophil heterogeneity and polarization regulation

Recent advances in single-cell RNA sequencing and spatial transcriptomics have reshaped the traditional understanding of neutrophil classification based on the N1/N2 paradigm. Emerging evidence indicates that neutrophils in MIRI are not a homogeneous pro-inflammatory population but rather consist of multiple subsets with distinct transcriptional profiles, functional states, and dynamic transition trajectories (121). For example, single-cell analyses have identified an MMP9^High neutrophil subset with enhanced NET-forming capacity, suggesting that this population may represent an important effector subset involved in inflammatory amplification and tissue injury (49). In addition, spatiotemporal omics and trajectory analyses further indicate that neutrophil fates are not fixed but undergo dynamic transitions during inflammatory progression, contributing to different processes including inflammation amplification, tissue damage, and subsequent repair (122). These findings suggest that neutrophil function should be understood as a continuous and dynamic spectrum rather than a simple N1/N2 binary classification.

Further studies have revealed that distinct neutrophil subsets exert opposing functions in MIRI. The Ccl3^+^ neutrophil subset exhibits pro-inflammatory characteristics, whereas the Ym-1^high neutrophil subset may exert protective effects by promoting reparative macrophage polarization (123). Another subset characterized by enhanced type I interferon signaling exerts cardioprotective effects in a STING-dependent manner. Neutrophil-specific STING deficiency worsens cardiac function and alters macrophage polarization, highlighting the distinct roles of different neutrophil subsets in injury and repair (124).

Beyond the intrinsic transcriptional states of neutrophils, local microenvironmental signals also participate in regulating their functional fate. Cardiomyocyte-derived miR-9-5p suppresses SOCS5 and SIRT1, activates the JAK2/STAT3 and NF-κB pathways, and drives neutrophils toward a pro-inflammatory N1 phenotype. Serum EV levels of miR-9-5p are associated with cardiovascular mortality in STEMI patients (125). In contrast, hypoxia-preconditioned cardiomyocytes secrete Stc1, which promotes Stat3 phosphorylation via calcium-sensing receptor and NOS2-mediated S-nitrosylation. This mechanism provides autocrine cardioprotection while paracrinely inducing neutrophil polarization toward a protective phenotype. Pericardial injection of recombinant Stc1 hydrogel extends the therapeutic window and improves long-term cardiac function (126). Together, these findings illustrate bidirectional paracrine regulation of neutrophil polarization by cardiomyocytes.

#### 4.5.2 Macrophage-related targets

Macrophage polarization regulates the recruitment of neutrophils and monocytes through chemokine signaling, forming intercellular positive feedback inflammatory loops. Following myocardial ischemia–reperfusion, Dectin-1 promotes M1 macrophage polarization and activates the IL-23/IL-1β/IL-17A axis, thereby aggravating myocardial injury; inhibition of Dectin-1 improves cardiac function (127).

TREM-1 is specifically expressed in CCR2^+^ macrophages and promotes recruitment of neutrophils and monocytes through CCL3 signaling. Deficiency of Trem1/3 prolongs survival of transplanted hearts (128). Upregulation of CLEC4E is associated with myocardial injury and functional impairment. Genetic deletion of CLEC4E attenuates myocardial damage, reduces infarct size, improves cardiac remodeling, and activates metabolic, antioxidant, and angiogenic pathways (129).

### 4.6 Novel drug delivery systems and biologics

With the deepening understanding of the mechanisms underlying MIRI, conventional pharmacological therapies face multiple limitations, including systemic side effects caused by nonspecific administration, insufficient drug accumulation in target organs, and poor *in vivo* instability of biomacromolecules.

In recent years, the development of novel drug delivery systems and biologics has provided innovative solutions to overcome these challenges. Nanoparticle-based delivery systems enable precise drug enrichment in injured myocardium through passive targeting (via increased vascular permeability at inflamed sites) or active targeting (via ligand–receptor recognition and cell membrane camouflage strategies). Biologics such as peptides, nanobodies, and modified mRNA offer high specificity and low immunogenicity, allowing precise regulation of key pathological molecular events. This section summarizes recent advances over the past few years in two major categories: nanotechnology-based targeted delivery systems and biologic/peptide-based therapeutic strategies (**Table 1**).

**Table 1:** Novel drug delivery systems and biologics for myocardial ischemia-reperfusion injury therapy.

Category	Drug/carrier	Mechanism of action	Key findings	Reference
Nanodelivery Systems	Neutrophil Decoys	Neutrophil-mimetic, H_2_O_2_-responsive controlled drug release	Targeted enrichment in damaged myocardium, utilizes neutrophil characteristics to protect cardiomyocytes, reduces infarct size	(130)
Nanodelivery Systems	SERCA Activator Nanoparticles	Utilizes neutrophil NETs for targeted drug delivery	NET-mediated targeting, significantly restores SERCA activity, improves cardiac function and remodeling	(131)
Nanodelivery Systems	Neutrophil Membrane-Coated Nanoparticles	Neutrophil membrane camouflage for active targeted delivery of allicin	Neutrophil membrane-mediated targeting, inhibits ferroptosis in cardiac microvascular endothelial cells, improves cardiac function	(132)
Nanodelivery Systems	Macrophage Membrane-Coated Nanoparticles	Targets S100A9 and evades lysosomal degradation	Reduces S100A9 levels, inhibits neutrophil infiltration and NETs formation, improves cardiac function	(133)
Biologics	RTP-026	Activates FPR2, inhibits neutrophil prolongation	Reduces infarct size by approximately 50%, decreases neutrophil and monocyte recruitment	(134)
Biologics	Modified mRNA Activating YAP	Activates YAP, inhibits TLR signaling pathway	Reduces cardiomyocyte necrosis and neutrophil infiltration, improves cardiac function, reduces scarring	(135)
Biologics	GV1001	Inhibits neutrophil infiltration	Reduces myocardial neutrophils, downregulates MPO activity and inflammatory factor levels	(136)
Biologics	Trimucrin	RGD disintegrin, inhibits platelet aggregation	Reduces infarct volume, myocardial injury markers, and neutrophil infiltration	(137)

In response to the mechanisms underlying neutrophil-mediated MIRI, multi-level therapeutic strategies have been developed and can be broadly categorized into six major approaches: targeting neutrophil recruitment and adhesion; inhibiting NETosis and effector functions; modulating intracellular signaling pathways; regulating mitochondrial function and oxidative stress; reshaping immune cell subsets and polarization; and developing novel drug delivery systems and biologics. These targets collectively form an integrated intervention network that spans the entire course of MIRI, from upstream blockades and effector suppression to signaling regulation, metabolic intervention, phenotypic reprogramming, and precision delivery. To facilitate comparison of the therapeutic targets, pharmacological agents, underlying mechanisms, experimental evidence, and potential clinical translational value of different treatment strategies, we further summarized representative therapeutic targets, drugs, mechanisms of action, evidence levels, experimental models, and translational potential in **Table 2**. However, significant challenges remain in translating these strategies into clinical practice, including optimal timing of intervention, cellular functional heterogeneity, the risk of immunosuppression, and inter-individual variability. The following section will focus on these key issues and discuss future directions for development.

**Table 2:** Comparison of therapeutic strategies for neutrophil-mediated myocardial ischemia–reperfusion injury.

Therapeutic strategy	Representative targets/drugs	Evidence level	Experimental models	Translational potential
Targeting neutrophil recruitment and adhesion	Nexinhib20, Navarixin, MKEY	Preclinical; clinical experience in related inflammatory diseases	Mouse MIRI models	High
Targeting NETosis and neutrophil effector functions	MLK3, PAD4, Colchicine	Preclinical + clinical association	Mouse MIRI models; STEMI patients	High
Targeting NLRP3 inflammasome	MCC950, Puerarin	Preclinical	Mouse MIRI models	Moderate
Targeting AMPK signaling	GEM, MK-3903	Preclinical	Mouse MIRI models	Moderate
Targeting mitochondrial dysfunction	MOTS-c, CBD	Preclinical	Mouse MIRI models	Moderate
Targeting oxidative stress	Silibinin, Bortezomib	Preclinical	Mouse MIRI models	Moderate
Targeting immune cell subsets and polarization	STING, Stc1	Preclinical	Mouse MIRI models	Early-stage
Novel drug delivery systems and biologics	Nanoparticles, hydrogels	Preclinical	Mouse MIRI models	Promising

## 5 Challenges and future perspectives

The clinical translation of neutrophil-targeted therapies for MIRI still faces several important challenges.

First, precise control of the therapeutic time window is critical for effective intervention. Neutrophils contribute to myocardial injury during the early phase of reperfusion through respiratory burst, NETosis, and the release of pro-inflammatory mediators. In contrast, during the later stages, they participate in inflammation resolution, angiogenesis, and tissue repair. Therefore, future therapeutic strategies should move beyond broad neutrophil suppression and instead adopt stage-specific modulation that limits detrimental neutrophil activation while preserving their physiological roles in host defense and tissue repair.

Second, target specificity and treatment safety remain major barriers to clinical application. Because neutrophils are indispensable components of the innate immune system, systemic inhibition may increase susceptibility to infection. Consequently, achieving localized and temporally controlled immunomodulation within the injured myocardium has become an important research objective. Emerging approaches, including nanoparticle-based drug delivery systems, inflammation-responsive carriers, and biologic agents, offer promising opportunities to improve drug accumulation at the site of injury while minimizing systemic adverse effects. In addition, growing evidence indicates that neutrophils are functionally heterogeneous, with distinct subsets exerting either detrimental or reparative functions during MIRI. Future studies should therefore focus on identifying and selectively targeting pathogenic neutrophil subsets while preserving protective populations.

Third, species differences and patient heterogeneity continue to hinder clinical translation. Most preclinical studies rely on young and otherwise healthy animal models, whereas patients with MIRI are often older and present with metabolic disorders, chronic inflammatory conditions, and multiple comorbidities. These factors can substantially influence neutrophil phenotype, inflammatory responses, and therapeutic efficacy. Accordingly, future investigations should employ disease models that more closely reflect clinical conditions and integrate circulating inflammatory biomarkers, immune phenotyping, and multi-omics approaches to facilitate patient stratification and precision therapy. Combination strategies that simultaneously target neutrophil recruitment, NETosis, inflammatory signaling, and cardiomyocyte-protective pathways may further enhance therapeutic efficacy.

Overall, the future development of neutrophil-targeted therapies is expected to shift from nonspecific functional inhibition toward spatiotemporally precise immunomodulation. The integration of advanced drug-delivery technologies, biomarker-guided patient stratification, and multi-target therapeutic strategies may accelerate the translation of experimental findings into clinical practice.
